# Liquid Membranes for Efficient Recovery of Phenolic Compounds Such as Vanillin and Catechol

**DOI:** 10.3390/membranes11010020

**Published:** 2020-12-28

**Authors:** Sandra Pavón, Luisa Blaesing, Annika Jahn, Ines Aubel, Martin Bertau

**Affiliations:** Institute of Chemical Technology, TU Bergakademie Freiberg, Leipziger Straße 29, 09599 Freiberg, Germany; sandra.pavon-regana@chemie.tu-freiberg.de (S.P.); luisa.blaesing@chemie.tu-freiberg.de (L.B.); annika.jahn@chemie.tu-freiberg.de (A.J.); ines.aubel@chemie.tu-freiberg.de (I.A.)

**Keywords:** vanillin, lignin monomer production, membrane technology, supported liquid membrane

## Abstract

Investigations were carried out to obtain different lignin monomers such as vanillin and catechol as efficiently as possible, to prevent side reactions e.g., during lignin degradation. Therefore, extraction experiments were performed to determine the influence of parameters such as initial pH in the aqueous phase, organic phases containing alcohols or solvating extractants, and monomer concentrations. Cyanex 923 (Cy923) and tri-n-butyl-phosphat (TBP) diluted in kerosene were the organic phases chosen to evaluate the transport of vanillin because of their high efficiencies (>76.8%) and suitability in membrane technologies. The most efficient vanillin transport was accomplished with Cy923, as > 90% of vanillin was transferred after 5 h. However, the permeability coefficient at carrier concentration of > 0.48 mol/L was influenced not only by the diffusion but also by the organic mixture viscosity. Thus, this concentration was used in the membrane experiment containing a mixture of vanillin and catechol in the feed phase. Catechol was transported about 7% faster to the receiving phase than vanillin, presumably due to its chemical structure. Side reactions were avoided using the current liquid membrane set-up, allowing the further industrial application of an entire process, which, e.g., recovers vanillin from enzymatic lignin conversion by membrane technology.

## 1. Introduction

Vanillin is the most important and frequently used flavoring agent worldwide, with a production volume of up to 20,000 tons per year [1]. Natural vanillin is obtained from the vanilla pod, where the extraction and preparation processes require up to nine days. The final product is of high quality and very aromatic, but with prices ranging from $2000/kg to $4000/kg [2] depending on the initial vanillin source. Owed to the high consumption, cultivation of natural vanilla cannot sufficiently supply global demand for vanillin, which is majorly used in the food, fragrance, cosmetic, and pharmaceutical industries. In fact, 97% of the vanillin is of synthetic origin, from substances such as coniferin, guaiacol, eugenol and lignin [1,2]. The increasing awareness of sustainable, healthy food and flavors has encouraged industry to access this valuable monolignol synthetically, under mild and almost natural conditions. Special attention is paid to biotechnological approaches, where natural vanillin extraction from lignin by microbial or enzymatic degradation is investigated. Besides vanillin, other aromatic substances such as guaiacol, acetovanillone, syringol, and catechol arise from lignin degradation. These are used in various fields, such as smoking flavor and sweet aroma in the food and fragrance industry or as fine chemicals in dyes, antiseptics, antioxidants as well as in other applications [3,4,5]. The high reactivity of vanillin renders it susceptible to oxidation to vanillic acid, though, which subsequently may decarboxylate to give guaiacol. *O*-demethylation of the latter results in catechol, which is further degraded by oxidative ring cleavage [6]. Although vanillin degrades rapidly, the microorganisms used in a biotechnical degradation of lignin may suffer from being inhibited, since vanillin as an aldehyde and acts toxic due to its high chemical reactivity [7,8]. To prevent such reactions and to obtain the target product vanillin in high yield and purity, separation of these substances has been investigated extensively [9,10]. However, selective separation of particular monomers often proves difficult, where structures are similar and chemically only poorly distinct. What renders the situation even more complex, is the possibility to convert the monomers and their degradation products into each other.

The clue to increase product selectivity and yield is preventing vanillin from reacting or being degraded, i.e., it has to be separated instantly from the reaction mixture once it has been formed. Different methods such as distillation, extraction, and chromatography have been investigated to separate monomers from lignin degradation after or during the process. The separation of vanillin during the reaction would be of great advantage since further degradation reactions by enzymatic and radical reactions are avoided, as well as the re-polymerization of the monomers among themselves or back to lignin-similar structures. By distillation [11] only very small amounts of the monomers can be separated, higher separation factors have been reported using a wide variety of extractants by extraction processes [10,12]. Generally, solvent extraction is one of the most common separation techniques to recover both metals and organic compounds on a large scale [13,14]. However, considering the low concentration of the main monolignols that constitute lignin, which can be obtained through enzymatic, thermal, chemical, and physical processes, membrane technology is more suitable because of its low operating costs and energy consumption. Liquid membranes have been used the recent years to treat individual phenolic or amino acid compounds in wastewater and food samples [15,16,17,18]. The carrier used as the liquid membrane has mostly been a mixture of an organic-solvent systems. Due to the introduction of solvents, the viscosity of the organic compound is reduced, which tends to promote the obtained transport rates. For instance, trioctylphosphine oxide (TOPO) or tri-n-butyl-phosphat (TBP) diluted in kerosene, were used to transport 65% of phenol and 64.5% of vanillin after 24 h, respectively by liquid membrane technology [1,19]. Within a variety of membrane types, the supported liquid membrane (SLM) is a promising technology as (i) extraction and stripping are performed simultaneously; (ii) the possibility of membrane leakage into the feed stream is avoided compared to the emulsion liquid membranes (ELM), and (iii) the organic phase consumption is reduced [20,21,22]. This low organic usage by SLM technology compared to liquid–liquid extraction offers presently one of the most significant improvements because it is in accordance with the current trend in “Green Chemistry” [1]. Among SLMs, the flat sheet configuration has been chosen here to investigate the transport of different aromatics. The flat sheet SLM (FSSLM) is based on a three-phase system with two aqueous solutions connected through an organic phase, which is immobilized in polymeric inert support, placed in between [20,23].

In the current work, the transport of an aromatic compound such as vanillin is accomplished using different organic carriers such as TBP, Cyanex 923 (Cy923, a mixture of straight-chain alkylated phosphine oxides) and compounds with a functional hydroxyl group. To choose the optimal parameters, which allow the successful transport of the monolignols, preliminary extraction experiments were carried out. The effect of the pH in the aqueous phase, of different organic extractants such as TBP, Cy923, butanol (BuOH) and decanol (DeOH), as well as of varying concentrations of vanillin and catechol on the extraction rate was investigated by liquid–liquid extraction. Once the optimal operating conditions had been fixed, the influence of the concentration of the carriers on the vanillin transport was investigated. Thus, the phenomenon controlling the transport was identified and a model equation, which relates the permeability to carrier concentration, was obtained. Finally, yet importantly, a mixture of vanillin and catechol was studied to investigate not only their transport but also the potential selective separation of the most valuable component such as vanillin and the possibility of avoiding re-polymerization when both are in the mixture. The satisfactory results obtained in the current work provide the basis for working with real samples after the enzymatic or thermal conversion of lignin and with hollow fiber membranes in further research.

## 2. Theoretical Background

The extraction reaction of each monomer (catechol and vanillin) with the four different extractants (BuOH, DeOH, TBP, and Cy923) [1,17] is depicted in Equations (1)–(4) as follows:

(1) $${\mathrm{MonOH}}_{\mathrm{aq}}+\bar{\mathrm{BuOH}}⇌\bar{\mathrm{MonOH}\cdot \mathrm{BuOH}}$$

(2) $${\mathrm{MonOH}}_{\mathrm{aq}}+\bar{\mathrm{DeOH}}⇌\bar{\mathrm{MonOH}\cdot \mathrm{DeOH}}$$

(3) $${\mathrm{MonOH}}_{\mathrm{aq}}+\bar{\mathrm{TBP}}⇌\bar{\mathrm{MonOH}\cdot \mathrm{TBP}}$$

(4) $${\mathrm{MonOH}}_{\mathrm{aq}}+\bar{\mathrm{Cy}923}⇌\bar{\mathrm{MonOH}\cdot \mathrm{Cy}923}$$

where MonOH refers to any of the two most representative monomers contained in lignin (catechol and vanillin).

The equilibrium constants, K_ex_ can be illustrated by Equations (5)–(8):

(5) $$K_{\mathrm{ex},\mathrm{BuOH}}⇌\frac{\left[\bar{\mathrm{MonOH}\cdot \mathrm{BuOH}}\right]}{\left[{\mathrm{MonOH}}_{\mathrm{aq}}\right]\cdot \left[\bar{\mathrm{BuOH}}\right]}$$

(6) $$K_{\mathrm{ex},\mathrm{DeOH}}⇌\frac{\left[\bar{\mathrm{MonOH}\cdot \mathrm{DeOH}}\right]}{\left[{\mathrm{MonOH}}_{\mathrm{aq}}\right]\cdot \left[\bar{\mathrm{DeOH}}\right]}$$

(7) $$K_{\mathrm{ex},\mathrm{TBP}}⇌\frac{\left[\bar{\mathrm{MonOH}\cdot \mathrm{TBP}}\right]}{\left[{\mathrm{MonOH}}_{\mathrm{aq}}\right]\cdot \left[\bar{\mathrm{TBP}}\right]}$$

(8) $$K_{\mathrm{ex},\mathrm{Cy}923}⇌\frac{\left[\bar{\mathrm{MonOH}\cdot \mathrm{Cy}923}\right]}{\left[{\mathrm{MonOH}}_{\mathrm{aq}}\right]\cdot \left[\bar{\mathrm{Cy}923}\right]}$$

The stripping process of each monomer from the organic phase can be successfully ensured by using a basic aqueous solution such as NaOH (Equations (9)–(12)).

(9) $$\bar{\mathrm{MonOH}\cdot \mathrm{BuOH}}+{\mathrm{NaOH}}_{\mathrm{aq}}⇌{\mathrm{MonONa}}_{\mathrm{aq}}+\bar{\mathrm{BuOH}}+H_{2}O$$

(10) $$\bar{\mathrm{MonOH}\cdot \mathrm{DeOH}}+{\mathrm{NaOH}}_{\mathrm{aq}}⇌{\mathrm{MonONa}}_{\mathrm{aq}}+\bar{\mathrm{DeOH}}+H_{2}O$$

(11) $$\bar{\mathrm{MonOH}\cdot \mathrm{TBP}}+{\mathrm{NaOH}}_{\mathrm{aq}}⇌{\mathrm{MonONa}}_{\mathrm{aq}}+\bar{\mathrm{TBP}}+H_{2}O$$

(12) $$\bar{\mathrm{MonOH}\cdot \mathrm{Cy}923}+{\mathrm{NaOH}}_{\mathrm{aq}}⇌{\mathrm{MonONa}}_{\mathrm{aq}}+\bar{\mathrm{Cy}923}+H_{2}O$$

To evaluate the extraction and stripping efficiencies of vanillin and catechol, the percentage of extraction and the percentage of stripping were calculated as described in Equations (13)–(14) considering that the volumes of both phases (V_organic_, V_aqueous_) are equal:

(13) $$\mathrm{Extraction}\;\left[\%\right]=\frac{{\left[\mathrm{MonOH}\right]}_{0}-{\left[\mathrm{MonOH}\right]}_{\mathrm{aq}}}{{\left[\mathrm{MonOH}\right]}_{0}}\cdot 100$$

(14) $$\mathrm{Stripping}\;\left[\%\right]=\frac{{\left[\mathrm{MonOH}\right]}_{\mathrm{str}}}{\left[\bar{\mathrm{MonOH}}\right]}\cdot 100$$

where ${\left[\mathrm{MonOH}\right]}_{0}$ and ${\left[\mathrm{MonOH}\right]}_{\mathrm{aq}}$ refer to the initial and the equilibrium concentration of each monomer in the aqueous phase. ${\left[\mathrm{MonOH}\right]}_{\mathrm{str}}$ is the equilibrium concentration of each monomer in the stripping phase and $\left[\bar{\mathrm{MonOH}}\right]$ is the concentration of each monomer in the loaded organic phase calculated by mass balance.

**Figure 1:**
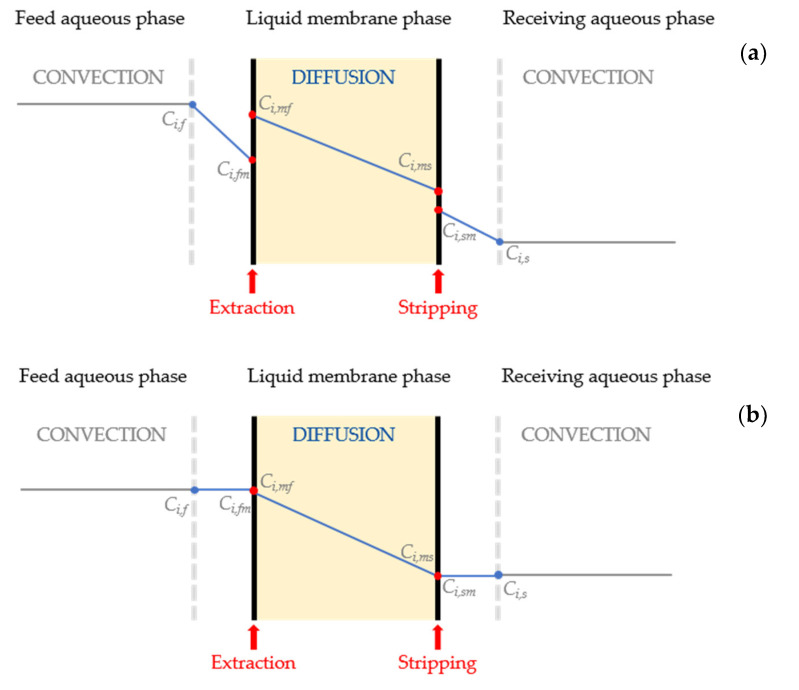
Concentration profile for the transport of the different monomers. (**a**) considering the three different resistances (diffusion on feed and stripping sides and through the membrane). (**b**) considering the diffusion through the liquid membrane as the limiting step.

Transport of the different monomers through the liquid membrane includes (i) diffusion resistance on the feed side; (ii) diffusion through the liquid membrane; and (iii) diffusion resistance on the stripping side (Figure 1a). However, the resistances on the feed and the stripping sides can be neglected due to the minimization of the boundary layer thickness because the stirrers in both cells are very close to the membrane. Thus, the diffusion through the liquid membrane is the limiting step of the transport process. Furthermore, it can be assumed that the extraction of the monomers using different carriers as well as the stripping reaction using NaOH take place instantaneously. The concentration profile for the different monomer transports is shown in Figure 1b.

As is well known, Fick’s first law controls the diffusion process and the flux through the SLM can be described by:

(15) $$J=-D\cdot \frac{d{\left[\mathrm{Monomer}\right]}_{f}}{\mathrm{dx}}$$

where J is the diffusion monomer molar flux (mol/s·m^2^), D is the diffusion coefficient (m^2^/s), x is the position (m) and [Monomer]_f_ is the concentration of each monomer in the feed phase (mol/L) at elapsed time (h).

Following the mathematical development of Pavón et al., the permeability coefficient, P (m/h) whose value refers to the speed of transport of each monomer from the feed phase to the stripping phase [20] can be determined by Equation (16):

(16) $$ln\frac{C_{i,f}}{C_{i,o,f}}=-P\cdot \frac{A}{V}\cdot t$$

where C_i,f_ and C_i,o,f_ are the concentration of each monomer in the feed phase and the initial concentration, respectively.

## 3. Materials and Methods

### 3.1. Materials

Catechol was obtained from Alfa Aesar by Thermo Fisher Scientific, Kandel, Germany. Sodium hydroxide, vanillin, kerosene, 1-decanol, TBP and 1-butanol were purchased from Sigma Aldrich Germany, Darmstadt, Germany. Citrate acid was supplied from Purux, Laaber, Germany and Cy923 was ordered from Solvay S.A. Brussels, Belgium.

### 3.2. Experimental Procedure

#### 3.2.1. Solvent Extraction

Solvent extraction experiments were conducted with an aqueous phase containing vanillin or catechol dissolved in citrate buffer (0.1 mol/L). Different parameters such as initial pH (from 3 to 5), different extractants (BuOH, DeOH, Cy923 and TBP) or monomer concentration (from 50 to 200 mg/L) were varied to investigate their influence on the extraction efficiency. 20 mL of the aqueous phase were equilibrated with an equal volume of organic phase in a separatory funnel at room temperature (25 ± 1 °C) using a horizontal mechanical shaker (model 3017, Gesellschaft für Labortechnik mbH, Burgwedel, Germany) at 150 rpm for 30 min. After separation of the two phases, the aqueous phase was removed and stored for further analyses. The monomer contained in the organic phase was stripped with 0.50 mol/L NaOH. To ensure the statistical certainty of the determined values, each experiment was performed three times.

#### 3.2.2. Transport

The transport experiments were conducted using an experimental set-up similar to the one depicted by Pavon et al. [20,24], in which two cylindrical compartment cells are connected by a lateral window where the FSSLM was placed. The FSSLM was prepared by soaking the polymeric inert support with the carrier solution and then wiping it with filter paper. The support used was a microporous polytetrafluoroethylene film (Fluoropore™ FHLP04700, Merck Millipore, MA, USA) and its characteristics are described in Table 1.

The feed solution was 100 mg/L of vanillin (or a mixture of vanillin and catechol) adjusted at pH 4 with 0.1 mol/L citrate buffer and 0.50 mol/L NaOH was used as receiving solution. The feed and the receiving cells were filled with 220 mL of the respective solutions, which were mechanically stirred at 1000 rpm at room temperature (25 ± 1 °C). Samples from each feed and stripping solution were taken periodically to determine the monomer concentration. All experiments were carried out in duplicate.

### 3.3. Analyses

The individual monomer concentrations were detected by Ultraviolet-visible spectrophotometry (UV-Vis, V-630, JASCO Deutschland GmbH, Pfungstadt, Germany). A spectrum between 250 and 600 nm was recorded and the respective maxima of substances at the corresponding pH values were observed, see Table 2.

Vanillin and catechol concentrations in the feed phase were determined a high-performance liquid chromatography (HPLC) Ultimate 3000 from Thermo Fisher Scientific, MA, USA and a nucleodur C18ec (250/4.6) column from Macherey–Nagel^®^, Düren, Germany. As the mobile phase, an acetonitrile-pure water mixture of 20:80 at a flow rate of 0.8 mL/min was applied. Detection was conducted using a diode array detector at a wavelength of 280 nm. For the analysis of both monomers in the receiving phase, a gel permeation chromatography (GPC) was used with a Thermo Fisher Scientific ion chromatography system with a UV detector ICS Series VWD of Dionex Thermo Fisher Scientific, MA, USA and viscometry detector of Polymer Standards Service (PSS) GmbH, Mainz, Germany. The eluent used was 0.1 mol/L NaOH with a flow rate of 1 mL/min at 60 °C. The columns used are MCX columns (1000 Å and 100,000 Å) from PSS GmbH, Mainz, Germany.

The water content of the organic phases was investigated using a Karl Fischer titrator (V20, Mettler Toledo, Gießen, Germany). An Ubbelohde viscometer at 20 °C (Jenaer Glaswerk Schott&Gen., Jena, Germany) was used to measure the viscosity of different organic phases. To obtain a density analysis of the organic phase, all solutions were first tempered at 20 °C for 30 min and afterward the measurements were carried out by triplicate using hydrometers.

## 4. Results and Discussion

### 4.1. Solvent Extraction

Considering the three main purposes of using membrane technologies: (i) determining vanillin and catechol transport, (ii) investigating their selective separation, and (iii) avoiding side reactions when both are in the mixture, the key parameters involved in the recovery process have to be investigated. In this sense, prior to the FSSLM experiments, the effect of the initial pH in the feed phase, the different extractants and the different monomer concentrations on the extraction efficiency had been studied by solvent extraction. This way, the operational range of these parameters was reduced, thus allowing for efficiently optimizing monomer transport when membrane technique is applied.

#### 4.1.1. Effect of Different Extractants and Initial pH

The initial pH value of the aqueous phase can take a significant effect on extraction. It depends on the extraction mechanisms involved and hence, the chemical reagent used for the extraction process. Although in the current research the enzymes have not been introduced in the system, the objective is developing an enzymatic conversion with a subsequent extraction stage to recover different monomers from lignin, especially vanillin, avoiding their re-polymerization. Despite the low pH values (1–2) reported by the literature to extract phenolics such as vanillin and phenol [1,23,25], the pH value was varied from 3 to 5 considering the aim of biological production of vanillin from lignin by direct enzymatic re-polymerization. As already mentioned, the focus during extraction is on vanillin, for which reason the optimal conditions were defined for this compound. However, the previous enzymatic conversions mainly led to catechol [3,26], thus the optimal extraction parameters obtained for vanillin recovery will be set and used for the study of the recovery of catechol.

**Figure 2:**
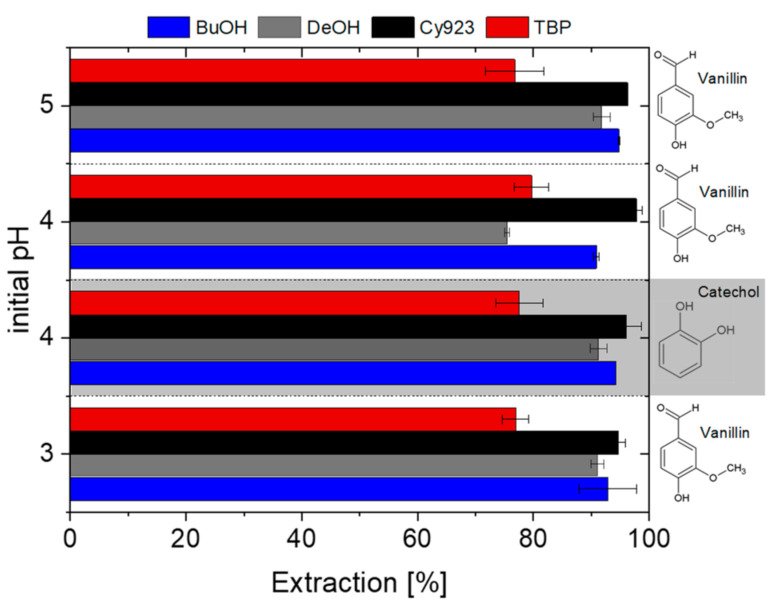
Effect of different initial pH values and extractants on the vanillin and catechol extraction.

Butanol (BuOH), decanol (DeOH), Cy923, and TBP were used to investigate the effect of these four extractants on monomer extraction efficiency. Both solvating extractants were diluted in kerosene and used with a molarity of 0.24 mol/L. Figure 2 shows the extraction efficiency for vanillin depending on the initial pH in the aqueous phase. No differences in the vanillin extraction efficiency within the pH range studied were observed since > 76.8% of this monomer was recovered using an initial pH of 3, 4, and 5. As expected, when solvating extractants or alcohols such as BuOH or DeOH are used, the pH value has no influence on the process since their reaction mechanisms are defined as the extraction of a neutral molecule (vanillin) [17]. Hence, the initial pH and the equilibrium pH were unchangeable. Therefore pH 4 was chosen as the optimal pH value for all subsequent experiments since laccases and peroxidases show the highest activity in a buffer solution in photometric determinations with 2,2′-Azino-bis(3-ethylbenzothiazoline)-6-sulfonate (ABTS) as the substrate [27,28] and highest product yields during lignin conversion. According to the current results and the literature suggestion, the initial pH was fixed to evaluate the extraction efficiency for catechol by varying the organic phase.

Comparing the extraction efficiency using the four selected organic compounds, it can be concluded that all these are able to extract vanillin from the citrate buffered aqueous phase under the working conditions with yields ≥ 76.8%. It can also be observed that BuOH, DeOH, as well as Cy923, provide higher efficiencies reaching up to 96.0% (aqueous phase (AP): pH 4; organic phase (OP): Cy923 0.24 mol/L) compared to TBP (76–78%). Similar trends were obtained for catechol recovery, as can be seen in Figure 2. Unfortunately, no significant differences were received and therefore, under the operating conditions used, there is no possibility of selective separation of both monomers. An increase of TBP concentration, a variation of the phase ratio or even a new extraction stage could be considered for achieving full recovery of vanillin and catechol.

A comparison with literature values for the extraction of catechol with these extractants proves difficult since not much has been published. Taking phenol as a similar extractant, vanillin was fully extracted (99%) using TBP diluted with different solvents such as hexane, decane, 2-octanol and kerosene [19]. However, the stripping of vanillin was in a range of 45–70% when decane and 2-octanol were used as extractants. The highest re-extraction efficiencies (75–85%) were obtained using 0.2 mol/L of NaOH when the organic mixture was 50% TBP-50% hexane or 50% TBP/50% kerosene. In this sense, the results of the current work are in accordance with the literature reaching stripping yields ~85%.

The loaded vanillin could be stripped (>98.7%) efficiently with aqueous NaOH. However, this was the case only for DeOH and Cy923, yet regardless of the initial pH (Table 3). These results are in accordance with previously reported research where NaOH solution served as stripping reagent for vanillin and phenol, too [29,30,31,32].

However, vanillin was re-extracted poorly with a yield ≤ 65% when BuOH was the organic phase. This fact is explained by its high solubility (77 g/L) [33] in water compared to the other three extractants. 22.0% was the determined water content in BuOH by titration, whereas in DeOH it was 4.5% and TBP/Cy923 it was < 1%. As a consequence, BuOH is hardly suited as an extractant. As it is soluble in the aqueous phase, there is no sufficient efficiency for monomer extraction purposes. Although stripping of Cy923 proved highly efficient with NaOH, emulsion formation was observed loaded (Vanillin-Cy923) and NaOH got in contact. These emulsions were stable for days and did not separate spontaneously. This effect has already been described in the literature, where the emulsion formation is mainly caused by the usage of NaOH [17]. Burghoff et al. [34] have described that no emulsions occurred when using Ba(OH)_2_. It has already been described that sodium ions or sodium phenolate form a polarized complex together with the oxygen of Cy923, which interacts in the aqueous phase like a surfactant and thus favors the emulsion formation [34,35]. Thus, the samples were completely centrifuged after the stripping stage to achieve clear phase separations.

To sum up, BuOH is considered unsuitable because of its high water solubility and water content in the organic phase. Therefore, the monomer recovery process is less efficient compared to the efficiencies achieved by using DeOH, Cy923, or TBP. Considering an application in liquid membrane extraction processes, the high water solubility constitutes a problem resulting in a progressive loss of BuOH in the feed phase, which in turn provokes slower transport values or even transport inhibition. From a technical application point of view, emulsion formation with Cy923 appears problematic at first sight. This issue can be solved easily, though, through centrifugation. On a larger scale, this would be realized in continuous operation with a decanter. In fact, the high vanillin efficiencies obtained are promising once the separation technique is changed to membranes technologies instead of solvent extraction.

#### 4.1.2. Effect of Different Monomer Concentrations

**Figure 3:**
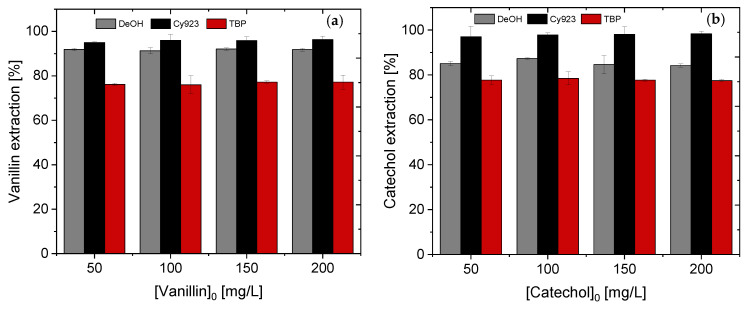
Effect of different monomer concentrations on the extraction yields of vanillin (**a**) and catechol (**b**).

During lignin degradation, the monomers are usually present in different concentrations. Jahn et al. [3] found that 1% vanillin and up to 6% catechol were generated from lignin after enzymatic conversion. With a 1 g/L lignin solution, this corresponds to concentrations of 10 mg/L and 60 mg/L, respectively. For the targeted vanillin separation, it is therefore, necessary to investigate whether different monomer concentrations affect extraction performance. The monomer concentration effect on the recovery efficiency was studied by conducting different experiments using a monomer range concentration from 50 to 200 mg/L and DeOH, Cy923, and TBP as organic phases. The extraction efficiencies are summarized in Figure 3 for both monomers. The results reveal that vanillin and catechol were extracted by these three extractants regardless of the monomer concentration within the studied range. The minimal extraction efficiency was 76.2% where vanillin was extracted by 0.24 mol/L TBP in kerosene (Figure 3a). However, as mentioned before, it can be improved by modifying other parameters such as the TBP concentration. However, Cy923 was able to almost completely extract both monomers using only one stage (> 96.0%). Furthermore, stripping efficiencies were higher when Cy923 was used, allowing the total mass balance to be adjusted to a single solvent extraction experiment (Table 4). In addition, the lowest extraction and stripping yields were experienced when DeOH was used, the solubility of which in water is problematic because of the extractant losses and therefore the extraction efficiency of vanillin and catechol decreases.

In conclusion, monomer concentration in the aqueous phase has no effect on the recovery efficiency of catechol and vanillin. Despite the lower monomer concentration range studied, the results are well in accordance with Yang et al. who demonstrated, too, that the phenol concentration (in a range of 1000–10,000 mg/L) is not a limiting parameter for extraction with TBP [36]. In this sense, it could be interesting to investigate a smaller range (< 50 mg/L) to verify that the concentration of the monomer has no influence on its extraction. Lower concentrations are also interesting since lignin conversion also results in lower concentrations of other monomers [3,26], such as syringol or guaiacol. Moreover, the solvating extractants seem to be promising using FSSLM instead of DeOH to avoid carrier loss problems because of the higher water solubility compared to TBP/Cy923. To ensure comparability with the literature and from our own experience in lignin conversion, the concentration of both monomers was fixed to 100 mg/L for the following experiments.

#### 4.1.3. Effect of Different TBP Concentrations on Vanillin Extraction

**Figure 4:**
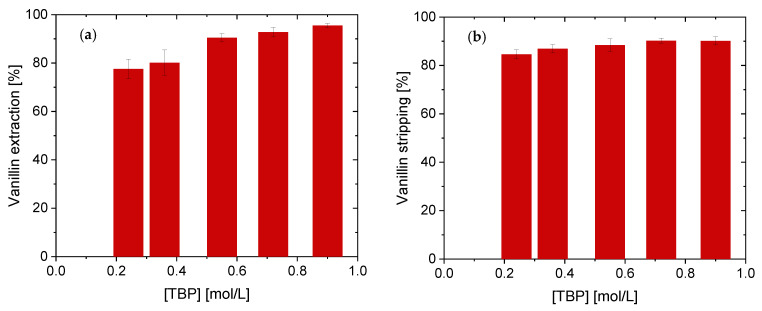
Effect of tri-n-butyl-phosphat (TBP) concentration on the vanillin extraction (**a**) vanillin stripping (**b**).

To improve the recovery efficiency of TBP for vanillin, the concentration of this solvating extractant diluted in kerosene increased up to 0.90 mol/L. The extraction and stripping yields are shown in Figure 4. As expected, the efficiency of vanillin recovery increases when the TBP concentration increased, too (Figure 4a).

Although 77.6% of vanillin was extracted using 0.24 mol/L of TBP, vanillin yield reached 95.3% by using the highest concentration studied (0.90 mol/L). This positive effect on the extraction process was also demonstrated by other researchers who used the same extractant on the vanillin extraction [1,19,37]. Re-extraction of vanillin loaded TBP with 0.50 mol/L NaOH gave efficiencies > 85% (Figure 4b). In the current work, 95.3% of vanillin has been extracted from the aqueous phase using 0.90 mol/L of TBP, whereas Zidi et al. reported 95% using twice as high TBP concentration [1]. This improvement is explained by the higher NaOH concentration in the stripping phase, which was 0.50 mol/L instead of 0.20 mol/L. However, at this NaOH concentration, vanillin is deprotonated to sodium vanillate. Considering the aim of applying this system to the membrane technology, VanOH·TBP cannot be transferred back into the feed phase once it has been transported to the receiving phase.

### 4.2. SLM experiments

According to the results obtained by using solvent extraction (SX), membrane experiments were carried out using the optimized parameters of initial pH in the feed phase (pH = 4), monomer concentration (100 mg/L) and the carriers which seem promising for an efficient and fast monomer transport (TBP and Cy923).

#### 4.2.1. Effect of Carrier Concentration on Vanillin Transport

**Figure 5:**
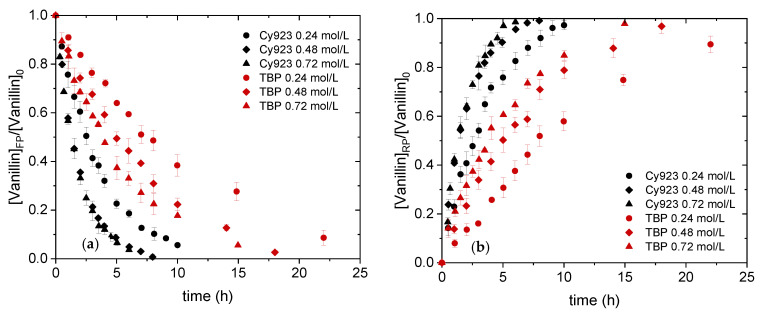
Effect of carrier concentration on the vanillin transport in the Feed phase (FP) (**a**) and in the receiving phase (RP) (**b**). FP: 100 mg/L vanillin in cb pH 4; RP: 0.50 mol/L NaOH.

The effect of Cy923 and TBP concentration in the organic membrane phase on vanillin transport was investigated in a range from 0.24 to 0.72 mol/L. Despite the higher vanillin extraction (95.3%) when 0.90 mol/L of TBP diluted in kerosene was used, the viscosity effect on the monomer transport has to be considered, too. Previously, reported studies suggest TBP concentrations ≤ 0.72 mol/L [1] because the transport flux decreases when a higher carrier concentration is used, which is down to the higher viscosity of the organic phase [38]. Figure 5 summarizes the results of the vanillin transport for both feed and receiving phases. It can be seen that the transport depends on the carrier concentration in the liquid membrane phase, since the formation of the complex species VanOH·TBP and VanOH·Cy923, respectively, in the liquid membrane are favored when the carrier concentration is increased as depicted in Equations (3) and (4). However, this complex species formation is not only dependent on the equilibrium constant of the reaction mechanisms, but also on the viscosity effect which is responsible for the impaired transport when too high.

Although both carriers, TBP and Cy923, were able to transport the vanillin from the feed to the receiving phase, the flux of vanillin transport was higher using Cy923 in the whole studied concentration range. 91.4% of vanillin was transported after 22 h using 0.24 mol/L TBP, whereas > 94.4% of this monolignol was obtained after 8 h in the receiving phase when Cy923 was used as a carrier. Increasing the carrier concentration to 0.72 mol/L, the vanillin was completely transported from the feed phase to the receiving phase after 5 h using Cy923. The results are similar to those reported in the literature using another kind of carrier such as palm oil, reaching transport efficiencies > 80% for several cresols after 5 h using the same membrane configuration (FSSLM) [23]. However, the phenol transport reported by Zidi et al. using tris-*n*-octyl phosphine oxide (TOPO) was slower, since ≤ 65% of phenol was transferred to the receiving phase [19]. This effect is explained by the differences in the structural characteristics of the support membranes (e.g., tortuosity, thickness and porosity) [20]. Thus, transport can be even more enhanced by using a hollow fiber module, reaching the full vanillin transport in 3 h [32]. This successful result is not depending on the compound transported but on the configuration of the liquid membrane. This is the reason similar results have been reported for phenol transport using both hollow fiber liquid membranes [17] and ELM [39].

#### 4.2.2. Permeability Coefficient Determination

**Figure 6:**
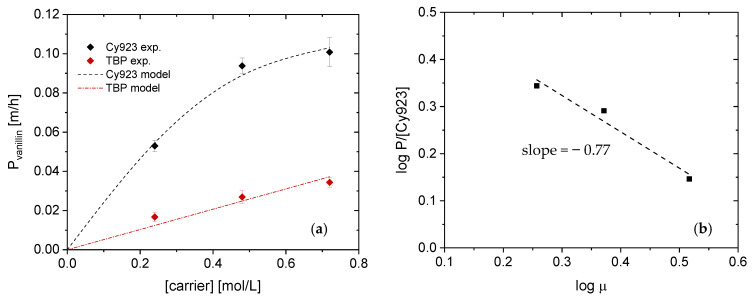
(**a**) Effect of the Cyanex 923 (Cy923) concentration on the vanillin permeability coefficient. (**b**) Effect of the dynamic viscosity on the diffusion coefficient using Cy923 as carrier. FP: 100 mg/L Vanillin in cb pH 4; RP: 0.50 mol/L NaOH.

Diffusion and viscosity effects on transport were examined in detail by determining the vanillin permeability coefficient (P) using TBP as well as Cy923. As seen in Figure 6a and as already shown in Section 4.2.1, the VanOH·Cy923 complex is transported faster than the VanOH·TBP complex. The permeability coefficients increase when the TBP concentration increases too, obtaining a linear dependence of carrier concentration and permeability coefficient. However, this linear proportionality was not observed when using Cy923, what is due to the slightly increased vanillin permeability coefficient with carrier concentrations > 0.48 mol/L.

As mentioned before, this fact is due to the viscosity increase. Diffusion is controlling vanillin transport through the liquid membrane using TBP as a carrier and thus, viscosity is not a parameter that restricts the flux of vanillin within the carrier concentration range studied. Therefore, the increase of the TBP concentration to achieve faster vanillin transport can be examined in further research. On the contrary, viscosity had a negative effect on the permeability coefficient of vanillin when the concentration of Cy923 is > 0.48 mol/L. Therefore, diffusion controls the transport process when the concentration of Cy923 is ≤ 0.48 mol/L. Therefore, the dynamic viscosity (µ) was determined by measuring the kinematic viscosity (ν) and the density of different organic solutions with TBP as carrier (Table 5).

The dynamic viscosity range within 0.24–0.72 mol/L of Cy923 is 1.81∙10^−2^ up to 3.29∙10^−2^ kg/mꞏs while the highest viscosity value is 1.68∙10^−2^ kg/mꞏs. An increase in the viscosity of the liquid membrane phase causes a diffusivity reduction of the vanillin complexes, as the diffusivity is inversely proportional to viscosity [40]. Nevertheless, the exact relationship between both parameters can be determined following the procedure previously reported [20]. Figure 6b depicts that the power for vanillin is −0.77 which is similar to the ones reported in the literature (−1 to −2/3) [20,41,42]. It is possible to obtain an equation that relates the permeability coefficient to the carrier concentration considering the viscosity effect, too (Equations (17) and (18)).

(17) $$P_{\mathrm{vanillin}}\;\left(m/h\right)=5.17\cdot 10^{-2}\cdot \left[\mathrm{TBP}\right]$$

(18) $$P_{\mathrm{vanillin}}\left(m/h\right)=3.59\cdot 10^{-1}\cdot \mu^{-0.77}\cdot \left[\mathrm{Cy}923\right]$$

Both equations allow for predicting vanillin transport as a function of TBP and Cy923 concentration. As expected, the permeability was higher when Cy923 was used as a carrier despite the negative effect of the viscosity using concentrations > 0.48 mol/L. Based on the results, 0.48 mol/L Cy923 was chosen as the carrier concentration to evaluate the transport of catechol and vanillin in a mixture.

#### 4.2.3. Transport and Permeability Coefficient of Vanillin and Catechol in Mixture

**Figure 7:**
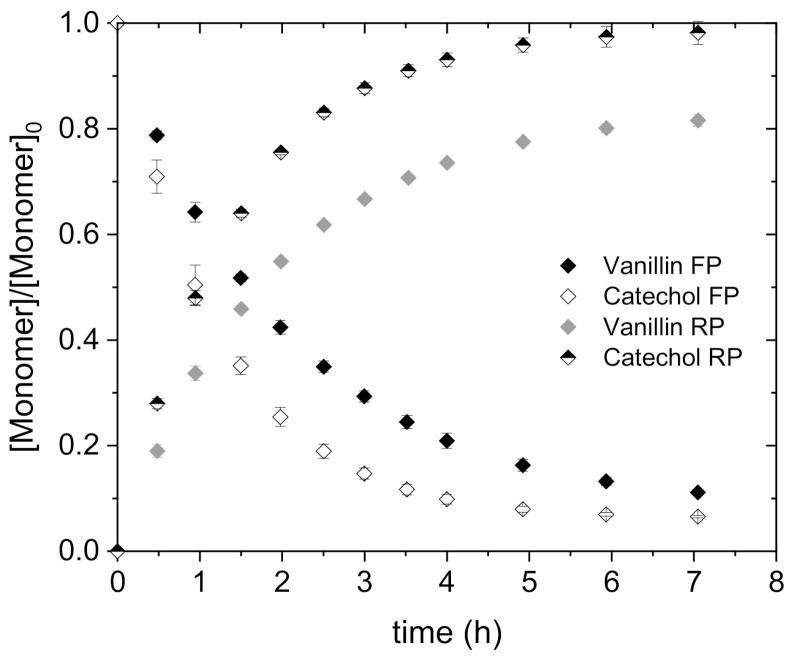
Transport of vanillin and catechol in both phases. FP: mixture of both monomers with a concentration of 100 mg/L each one in citrate buffer pH 4; OP: 0.48 mol/L Cy923; RF: 0.50 mol/L NaOH.

Once the transport and vanillin permeability coefficients were evaluated by FSSLM, a mixture of two monomers was used as feed phase to evaluate their potential of selective separation as well as to avoid the problem of side reactions during the time. The feed phase contained a mixture of vanillin and catechol in citrate buffer at pH 4 with 100 mg/L monomer concentration. 0.50 mol/L NaOH and 0.48 mol/L Cy923 were used as receiving phase and carrier in the liquid membrane phase, respectively. The concentration of both monomers in feed and receiving phases were analyzed by using two different techniques due to the different pH values in both phases. HPLC was able to measure the vanillin and catechol concentration in the feed phase whereas GPC is equipped to determine the monomer concentration in the receiving phase because of the strong alkaline range. The transport results are depicted in Figure 7 for both monolignols. Due to the use of both different analytical techniques, the exact mass balance was not reached yet.

Vanillin and catechol were transferred from the feed to the receiving phase after 7 h. The trend of vanillin transport was similar to that obtained in the individual FSSLM experiment using 0.48 mol/L. It can be shown that vanillin transport is 7% slower than catechol, so that the maximum concentration of vanillin in the receiving phase is ≤ 83.7% after 5 h. This behavior can be explained by the solubility of the substances and the pK_a_ value. Vanillin has a pK_a_ value of 7.4 and a solubility of 10 g/L in water, whereas catechol has a pK_a_ value of 9.6 and a solubility of 451 g/L. Thus, the affinity of vanillin to the organic membrane phase is higher than that of catechol, which results in slower transport through this organic phase. Therefore, the diffusion coefficient of catechol is higher than that of vanillin, resulting in a faster catechol transport after 7 h with 93.4%. In addition, the pKas value shows that the stripping reaction using NaOH (pH 14) with catechol is favored. By adjusting the pH value, it might be possible to influence the selectivity during separation of the monomers, since vanillin, for example, is better converted in the range of pH 67 [10]. However, the transport differences were not high enough to consider an effective selective separation of these monomers. The use of the stream obtained after enzymatic conversion as feed phase could be a promising alternative in further research to investigate the selective separation, since the concentration of both monomers differs considerably, being the conversion of catechol in a range from 5 to 7% higher than vanillin [3,26]. Therefore, the diffusion coefficient differences between these monolignols could allow the complete catechol transport avoiding the co-transport of vanillin.

Permeability coefficients for both monomers were determined by Equation (16). 1.11∙10^−1^ m/h was the obtained permeability coefficient of catechol whereas for vanillin it is 7.41∙10^−2^ m/h. In accordance with the above regarding the faster catechol transport, the P_catechol_ was 50% higher than the P_vanillin_. Despite that the initial pH in the aqueous phase had no influence on the vanillin extraction due to the use of solvating extractants such as TBP and Cy923, other parameters should be considered and modified to reach the selective separation. For instance, it can be possible using a different kind of carrier or varying the pH in the receiving phase, since the monomers differ in their pK_a_ values (pK_a(vanillin)_ = 7.4 and pK_a(catechol)_ = 9.45). Mante et al. have tried to extract various phenols with different pH values due to differences in the pK_a_ values [10]. Starting from alkaline range, the phase was acidified using HCl after the extraction with methyl-*tert*-butyl ether (MTBE) and thus different fractions were obtained during the extraction allowing further separation. Catechol was extracted at pH 9 and vanillin at pH 6. Based on these reported results, it can be concluded that fractionated extraction is possible. Different concentrations of NaOH were used to allow a selective extraction of phenolic components from bio-oil [9]. By increasing the concentration of NaOH up to 2 mol/L the separation of catechol and vanillin, among others, was improved. In addition, using < 0.1 mol/L NaOH, catechol was still better to be separated than vanillin. In conclusion, selective separation of vanillin and catechol from lignin via liquid membranes is promising when pH values and receiving phase concentration are modified.

**Figure 8:**
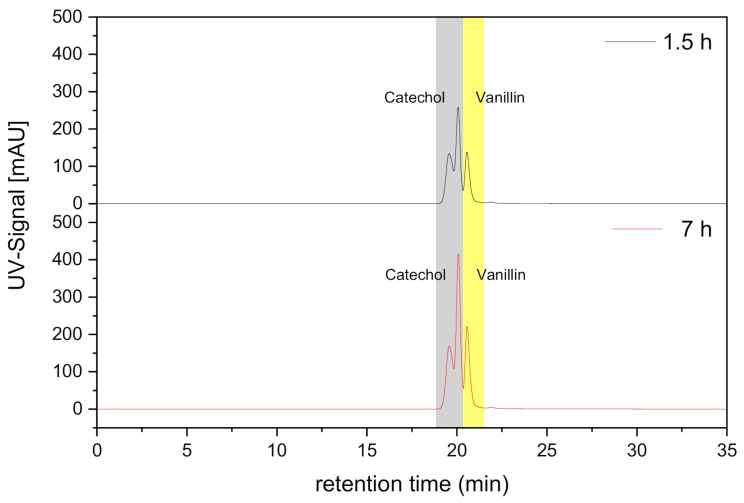
GPC spectra of the mixture vanillin-catechol in the receiving phase after 1.5 and 7 h. FP: mixture of both monomers with a concentration of 100 mg/L each one in citrate buffer pH 4; OP: 0.48 mol/L Cy923; RF: 0.50 mol/L NaOH.

Furthermore, the possible side reactions of vanillin and catechol in the receiving phase were evaluated by analyzing two samples (after 1.5 and 7 h) with GPC and comparing both chromatograms. As can be seen in Figure 8, the signals of both monomers are at the same retention time in both samples in different time periods. Thus, it can be confirmed that no side reactions of the monomers with each other have taken under the working conditions used in the current research using liquid membranes with flat sheet configuration. This would be an advance in lignin conversion since side reactions often occur there in the form of re-polymerization of the monomers, dimers, etc. in the system.

## 5. Conclusions

The transport of single vanillin, as well as a mixture containing vanillin and catechol, was investigated by using FSSLM to evaluate the potential selective separation and avoid side reactions once these monolignols are transferred to the receiving phase. To find the optimal parameters for both purposes, SX experiments were carried out identifying the influence of different factors such as initial pH, organic phase mixtures and monomer concentration on the extraction efficiency. Both monomers were extracted from the aqueous phase regardless of the initial pH and their initial concentration. However, the further goal is developing a complete process chain combining enzymatic conversion and subsequent SX or SLM technique which allows the individual vanillin recovery from lignin. In this sense, the chosen optimal pH was 4 considering the highest conversion in citrate buffer under these conditions reported previously [3]. Despite the high vanillin extraction using BuOH (> 90%), it was solubilized into the stripping phase due to its high water content (miscible). Although this problem was not observed using DeOH, Cy923 and TBP were the carriers chosen to investigate the monomer transport through liquid membranes to ensure the industrial feasibility with a membrane module scale-up.

The FSSLM experiments demonstrated how fast vanillin was transported from the feed to the receiving phase using Cy923 compared to TBP, reaching 96.4% after 6 h at 0.72 mol/L. The transport process was controlled by the diffusion with ≤ 0.48 mol/L Cy923 and the viscosity > 0.48 mol/L. However, the diffusion was the phenomena responsible for the vanillin transport using TBP within the carrier concentration range studied. It has been demonstrated the power of the viscosity when Cy923 was used. Furthermore, vanillin permeability coefficients were determined for both solvating extractants obtaining a model equation that predicts its transport as a function of the carrier concentration (P_TBP_ = 5.17∙10^−2^ m/h) or carrier concentration and viscosity (P_Cy923_ = 3.59∙10^−1^ m/h). In accordance with these results, 0.48 mol/L of Cy923 was used as the best choice to investigate the transport of vanillin and catechol in the mixture to evaluate their potential selective separation as well as the re-polymerization problem along time. Catechol was transferred 7% faster than vanillin due to the higher affinity to the NaOH phase resulting from the pK_a_ value and the solubility of catechol, reaching 93.4% of transport after 7 h. P_catechol_ was 11∙10^−1^ m/h whereas for vanillin it amounts to 7.41∙10^−2^ m/h. Although the selective separation was not achieved yet, it will be examined in further research modifying the initial pH and the receiving solution according to the successfully reported studies [9,10]. Nevertheless, side reactions were avoided using the current membrane set-up since the GPC chromatogram showed no differences during the time. This knowledge will be applied for real streams coming from lignin conversion to develop a process that allows vanillin recovery while avoiding re-polymerization by combining enzymatic reaction and membrane technology.

## Figures and Tables

**Table 1:** Characteristics of the microporous polytetrafluoroethylene film (Fluoropore™ FHLP04700).

Parameter	Value
Diameter (cm)	4.7
Pore diameter (µm)	0.45
Thickness (µm)	150 *
Effective area (m^2^)	11.4
Porosity (%)	85

* including 100 µm of the polyethylene grid.

**Table 2:** Wavelengths of the absorption maxima of investigated monomers vanillin and catechol at 0.1 mol/L citrate buffer (cb) pH 4 and 0.50 mol/L NaOH pH 14.

Monomers	Wavelength in cb pH 4 [nm]	Wavelength in NaOH pH 14 [nm]
Vanillin	280	345
Catechol	276	318

**Table 3:** Stripping efficiencies of vanillin and catechol using different organic phases (the colored efficiencies correspond to catechol).

	Stripping Efficiency [%]							
Initial pH	BuOH		DeOH		Cy923		TBP	
3	64.9 ± 4.45		99.8 ± 0.98		99.2 ± 2.14		85.2 ± 1.33	
4	64.9 ± 4.98	83.1 ± 4.15	99.9 ± 3.38	99.9 ± 3.11	98.7 ± 2.14	94.6 ± 2.24	84.6 ± 1.88	84.0 ± 1.93
5	61.6 ± 4.84		99.7 ± 3.43		99.2 ± 4.01		85.0 ± 1.42	

**Table 4:** Stripping efficiencies of vanillin and catechol using different monomer concentrations (the colored efficiencies correspond to catechol).

	Stripping Efficiency [%]					
Monomer Concentration [mg/L]	DeOH		Cy923		TBP	
50	97.9 ± 1.13	89.9 ± 1.80	96.3 ± 2.86	87.9 ± 1.01	99.9 ± 0.53	96.6 ± 3.57
100	99.9 ± 2.08	99.9 ± 2.61	99.9 ± 2.14	92.7 ± 2.28	91.3 ± 1.88	85.2 ± 3.94
150	96.9 ± 2.21	80.7 ± 1.60	99.9 ± 1.90	82.6 ± 2.00	97.6 ± 2.65	95.7 ± 2.18
200	99.9 ± 2.52	91.0 ± 1.30	96.8 ± 1.93	80.6 ± 1.50	94.4 ± 1.35	92.9 ± 3.20

**Table 5:** Physical properties of different organic solutions which contains TBP diluted in kerosene.

Carrier	Concentration [mol/L]	Density * [kg/m^3^]	*ν* * [10^−6^·m^2^/s]	µ [10^−2^·kg/m·s]
Cy923	0.24	803	2.25	1.81
	0.48	814	2.89	2.35
	0.72	828	3.98	3.29
TBP	0.24	806	1.90	1.53
	0.48	812	1.92	1.56
	0.72	838	1.99	1.68

* Density and cinematic viscosity were measured at 20 °C.
